# Convergence of a diabetes mellitus, protein energy malnutrition, and TB epidemic: the neglected elderly population

**DOI:** 10.1186/s12879-016-1718-5

**Published:** 2016-07-26

**Authors:** Sonia Menon, Rodolfo Rossi, Leon Nshimyumukiza, Aibibula Wusiman, Natasha Zdraveska, Manal Shams Eldin

**Affiliations:** 1 International Centre for Reproductive health, Ghent University, LSHTM Alumni, Ghent, Belgium; 2 CDC Foundation, Atlanta, USA; 3 LSHTM Alumni, Beirut, Lebanon; 4 Department of Social and Preventive Medicine, Laval University-Faculty of Medicine, Quebec, Canada; 5 Department of Epidemiology, Biostatistics, and Occupational Health, McGill University, Montreal, Canada; 6 Department of Clinical Pharmacy, Saints Cyril and Methodius University of Skopje (Alumni), Skopje, Republic of Macedonia; 7 Médecins Sans Frontières, Paris, France

## Abstract

**Background:**

On a global scale, nearly two billion persons are infected with Mycobacterium tuberculosis. From this vast reservoir of latent tuberculosis (TB) infection, a substantial number will develop active TB during their lifetime, with some being able to transmit TB or Multi-drug- resistant (MDR) TB to others. There is clinical evidence pointing to a higher prevalence of infectious diseases including TB among individuals with Diabetes Mellitus (DM). Furthermore, ageing and diabetes mellitus may further aggravate protein-energy malnutrition (PEM), which in turn impairs T-lymphocyte mediated immunologic defenses, thereby increasing the risk of developing active TB and compromising TB treatment. This article aims to a) highlight synergistic mechanisms associated with immunosenescence, DM and PEM in relation to the development of active TB and b) identify nutritional, clinical and epidemiological research gaps.

**Methods:**

To explore the synergistic relationship between ageing, DM, tuberculosis and PEM, a comprehensive review was undertaken. The MEDLINE and the Google Scholar databases were searched for articles published from 1990 to March 2015, using different MESH keywords in various combinations.

**Results:**

Ageing and DM act synergistically to reduce levels of interferon gamma (IFN- γ), thereby increasing susceptibility to TB, for which cell mediated immunity (CMI) plays an instrumental role. These processes can set in motion a vicious nutritional cycle which can predispose to PEM, further impairing the CMI and consequently limiting host defenses. This ultimately transforms the latent TB infection into active disease. A clinical diagnostic algorithm and clinical guidelines need to be established for this population.

**Conclusion:**

Given the increase in ageing population with DM and PEM, especially in resource-poor settings, these synergistic tripartite interactions must be examined if a burgeoning TB epidemic is to be averted. Implementation of a comprehensive, all-encompassing approach to curb transmission is clearly indicated. To this end, clinical, nutritional and epidemiological research gaps must be addressed without a delay.

## Background

TB infection occurs when a susceptible person inhales droplets containing *Mycobacterium tuberculosis* bacteria, which travel through the respiratory tract to the alveoli. In most patients, host’s immune response limits the propagation of TB infection, resulting in an asymptomatic, non-transmissible localized infection that may remain in the body for many years, if not forever.

One in three people in the world has latent tuberculosis [1]. In 2009, approximately 9 million new cases of active TB were diagnosed and 1.7 million persons succumbed to the disease [2]. An additional challenge to TB control efforts is the global increase in multi-drug resistant TB (MDR-TB), defined as TB caused by strains resistant to at least isoniazid and rifampin. In 2013, the World Health Organization (WHO) reported that 3.6 % of the new cases and 20.2 % of the previously treated cases had MDR-TB [3].

Concurrently, diabetes mellitus (DM) is burgeoning as a worldwide chronic health condition, which can be attributed to increases in obesity, changing patterns of diet and physical activity as well as ageing [4, 5]. According to WHO estimates, there are currently 347 million people worldwide affected by DM, [6] and by 2030 its prevalence is projected to increase by 50 % [6]. 3/4 of diabetic patients live in low-income countries [7–9]. It is now well-established that cellular immune responses mediated by T cells and macrophages play a major role in the defense against TB [10]. In particular, the Th1 cytokine interferon (IFN)-γ is considered a principal mediator of protective immunity against TB [11, 12]

DM is a clinical syndrome associated with deficiency of insulin secretion or resistance to its actions. Apart from the classical micro and macrovascular complications of the disease, DM has been associated with reduced T cell response and neutrophil functional activity as well as humoral immunity disorders, [13, 14] which in turn compromises the protective role that cellular immune response plays against TB. Consequently, DM patients show increased susceptibility to infections, notably TB, compared to individuals without DM [2, 15].

Some studies have shown that TB/DM comorbidity is common, both in low-income and high-income countries. [2, 16]. A 2008 systematic review of literature which identified 13 age-adjusted, quantitative, observational studies in North America, UK, Russia, Mexico, Korea, Taiwan and India reported a relative risk of TB in DM patients of 3.1 in cohort studies, with odds ratios ranging from 1.16 to 7.83 in case control studies [17]. An epidemiological model indicated that in India DM might account for nearly 15 % of pulmonary tuberculosis (PTB) cases [18].

In diabetic patients, pulmonary TB may progress rapidly and hence requires a high index of suspicion in the diagnostic phase [19].

Furthermore, the rising number of ageing diabetic population at risk for TB represents a worldwide health threat. People aged 60 and older make up over 11 % of the global population and by 2050 that number is expected to rise to about 22 % [20]. By 2050, 4 out of 5 people over 60 will live in developing countries [21]. In this population group, approximately 90 % of TB cases are due to reactivation of primary infection [22].

Ageing is associated with a decline in T cell proliferation and reduced synthesis of interferon gamma (IFN-γ), [23] which compromises body’s protective defenses against TB. In turn, by compounding the decrease in IFN – γ DM predisposes the ageing patient to infections where cell-mediated immunity plays a pivotal role, such as tuberculosis.

Moreover, ageing and DM act synergistically and further aggravate protein-energy malnutrition (PEM), which is common in chronic disease states and is associated with increased morbidity and mortality [24]. This tripartite interaction additionally impairs T-lymphocyte mediated immunologic defenses, increasing the risk of certain infectious diseases [25]. According to the WHO, the number of people with TB attributable to PEM may exceed the number of people whose TB develops secondary to HIV infection, smoking or DM [26]. The negative impact of undernutrition on cell-mediated immunity is well documented [25, 27–29]. Malnutrition and infection interact with each other synergistically. Recurrent TB may cause loss of body nitrogen and worsened nutritional status. The resulting malnutrition may in turn increase the susceptibility to recurrent infection.

Using data from existing literature, this article aims to highlight the synergistic mechanisms associated with immunosenescence and DM in relation to development of active TB. This includes the possible association between ageing and development of TB and/or PEM and the possible association between TB and PEM. The possibility of treatment for latent TB in the ageing population with DM is also explored.

Finally, this paper aims to identify the clinical and epidemiological research gaps which need to be addressed in order to curb transmission.

## Method

To explore the synergistic relationship between ageing, DM, tuberculosis and PEM, the MEDLINE and the Google Scholar databases were searched for articles published from 1960 to March 2015. The following keywords were identified using medical subject headings and truncations:

### Synergistic biological mechanisms between DM, ageing TB population and PEM

#### Ageing and TB

("ageing"[MeSH Terms] OR "ageing"[All Fields]) AND ("tuberculosis"[MeSH Terms] OR "tuberculosis"[All Fields]) yields 523 results

### Ageing and DM

("ageing"[MeSH Terms] OR "ageing"[All Fields]) AND ("diabetes mellitus"[MeSH Terms] OR ("diabetes"[All Fields] AND "mellitus"[All Fields]) OR "diabetes mellitus"[All Fields]) yields 8131 results

### Ageing and PEM

("ageing"[MeSH Terms] OR "ageing"[All Fields]) AND ("protein-energy malnutrition"[MeSH Terms] OR ("protein-energy"[All Fields] AND "malnutrition"[All Fields]) OR "protein-energy malnutrition"[All Fields] OR ("protein"[All Fields] AND "energy"[All Fields] AND "malnutrition"[All Fields]) OR "protein energy malnutrition"[All Fields]) yields 363 results

### PEM and TB

("protein-energy malnutrition"[MeSH Terms] OR ("protein-energy"[All Fields] AND "malnutrition"[All Fields]) OR "protein-energy malnutrition"[All Fields] OR ("protein"[All Fields] AND "energy"[All Fields] AND "malnutrition"[All Fields]) OR "protein energy malnutrition"[All Fields]) AND ("tuberculosis"[MeSH Terms] OR "tuberculosis"[All Fields] yields 91 results

### BMI and TB

BMI[All Fields] AND TB[All Fields] yields 2666 results.

### Treatment of latent TB in ageing population with DM

("therapy"[Subheading] OR "therapy"[All Fields] OR "treatment"[All Fields] OR "therapeutics"[MeSH Terms] OR "therapeutics"[All Fields]) AND ("latent tuberculosis"[MeSH Terms] OR ("latent"[All Fields] AND "tuberculosis"[All Fields]) OR "latent tuberculosis"[All Fields])) AND ("ageing"[MeSH Terms] OR "ageing"[All Fields])] yields 19 results

### Diabetes mellitus and multi drug resistant TB

("diabetes mellitus"[MeSH Terms] OR ("diabetes"[All Fields] AND "mellitus"[All Fields]) OR "diabetes mellitus"[All Fields]) AND multi [All Fields] AND ("drug resistance"[MeSH Terms] OR ("drug"[All Fields] AND "resistance"[All Fields]) OR "drug resistance"[All Fields] OR ("drug"[All Fields] AND "resistant"[All Fields]) OR "drug resistant"[All Fields]) AND TB. [All Fields] yields 19 results

### Diabetes mellitus and multi-drug resistant TB treatment

("diabetes mellitus"[MeSH Terms] OR ("diabetes"[All Fields] AND "mellitus"[All Fields]) OR "diabetes mellitus"[All Fields]) AND multi[All Fields] AND ("drug resistance"[MeSH Terms] OR ("drug"[All Fields] AND "resistance"[All Fields]) OR "drug resistance"[All Fields] OR ("drug"[All Fields] AND "resistant"[All Fields]) OR "drug resistant"[All Fields]) AND TB[All Fields] AND ("therapy"[Subheading] OR "therapy"[All Fields] OR "treatment"[All Fields] OR "therapeutics"[MeSH Terms] OR "therapeutics"[All Fields]) yields 15 results

### DM and PEM

"diabetes mellitus"[MeSH Terms] OR ("diabetes"[All Fields] AND "mellitus"[All Fields]) OR "diabetes mellitus"[All Fields]) AND ("protein-energy malnutrition"[MeSH Terms] OR ("protein-energy"[All Fields] AND "malnutrition"[All Fields]) OR "protein-energy malnutrition"[All Fields] OR ("protein"[All Fields] AND "energy"[All Fields] AND "malnutrition"[All Fields]) OR "protein energy malnutrition"[All Fields]) yields 164

Bibliographic search included WHO policy papers, personal communication, original articles and review articles written in English, French and Spanish. The following retrieved studies were included: longitudinal studies, randomized controlled trials, reviews or other comparative studies. Case reports were excluded. The included studies were then reviewed by two authors, and major findings reported. No ethical approval was required as this is a comprehensive paper without primary data collection.

## Results

Of the studies retrieved from the above-mentioned electronic database, 13 epidemiological studies and 6 (systematic reviews)/meta-analyses were included in this review. They are summarized in the Table 1 below:

**Table 1:** Table summarizing the findings of the studies used in this review

First author	Year of publication	Study design and sample size	Main exposure(s) of interest	Main outcome(s) of interest	Main results and Remarks
Peleg AY	2007	Literature review	Glycaemic control	Risk of common community acquired infections	Further research is needed to improve understanding of the role of diabetes and glycaemic control in the pathogenesis and management of community and hospital acquired infections
Leung CC	2008	Cohort study 42,116 clients aged 65 years or more,	Diabetes mellitus	TB	Among diabetic subjects, higher risks of active, culture-confirmed, and pulmonary but not extrapulmonary tuberculosis were observed, with baseline hemoglobin A1c ≥7 % (vs. <7 %)
Dick Menzies	2011	Review article	LTBI	TB	LTBI therapy should be given only to those with positive tests for LTBI. Underutilized, particularly in LMIC
Matthew J. Magee	2014	cohort of 1366 adult patients	DM	MDR TB	DM did not impact culture conversion rates in a clinically meaningful way, but smoking did.
Holt PR	2001	Review/Perspective	Elderly population	Malabsorption	Nutrition may be compromised rapidly by the reduction in food intake or malabsorption that accompanies many of the conditions that cause diarrhea in the elderly
Cruz-Hervert LP	2012	Cross sectional study of 893 65 years of age or older.	65 years of age or older	Clinical and epidemiological consequences of pulmonary tuberculosis	Untimely and difficult diagnosis and a higher risk of poor outcomes even after treatment completion emphasize the need for specific strategies in this vulnerable group.
J. Peter Cegielski	2012	Cohort 1982–1992 of 14,189 adults	BMI	TB	Population's nutritional profile is an important determinant of TB incidence.
Nyadzayo	2014 (still in press)	Cohort study 410 adults	TB	Recovery from moderate malnutrition	Moderately malnourished adults are less likely to recover their nutritional status compared to non-TB patients when under supplementary treatment
Kurbatova, E. V	2012	Cohort study of 1416 adults in 5 countries	predictors of initial sputum culture conversion in MDR TB treatment		Lower but not significant *unadjusted* rate of sputum culture conversion among patients with DM
Matthew J. Magee	2014	Cohort study of 1,366 adult patients in Georgia	MDR TB treatment in DM patients	culture conversion among patients with multidrug-resistant (MDR)-TB	In adjusted analyses, DM did not impact culture conversion rates in a clinically meaningful way
María Eugenia Jiménez-Corona	2013	Cohort study of 1262 patients with pulmonary TB in Mexico	Patients with DM	clinical consequences of pulmonary tuberculosis	Patients with DM and pulmonary TB had more severe clinical manifestations, delayed sputum conversion, a higher probability of treatment failure and recurrence
Meghan A Baker	2011	Systematic review and meta-analysis.	quantitative summary evidence for the impact of diabetes on tuberculosis outcomes		DM increases the risk of treatment failure and death combined, death and relapse among patients with tuberculosis.
Christie Y Jeon	2008	13 observational studies (*n* = 1,786,212 participants) with 17,698 TB cases	Patients with DM	Active TB disease	Meta-analysis shows that DM increases the risk of TB, regardless of different study designs, background TB incidence or geographic region of the study.
Stevenson CR	2007	Review	Patients with DM	Active TB disease	All studies identified statistically significant associations, with a 1.5- and 7.8-fold increase in risk or odds of TB in diabetic patients. Inadequate adjustment of potential major confounders.
Nijland HM	2006	1 prospective pharmacokinetic study (*n* = 17 adult patients	Patients with TB-DM comorbidity	Effect on plasma rifampicin levels	Study showed 53 % lower rifampicin exposure (AUC_0–6 h_) in TB-DM patients, compared to TB only patients.
Meghan A Baker	2012	Prospective study	Patients with DM	Active TB and severe TB	The risk of developing tuberculosis increased among those with increasing diabetes severity.
Brendan K. Podell,	2012	60 guinea pigs were randomly assigned to Mtb infected and sucrose-fed (*n* = 20), Mtb infected and water-fed (*n* = 20), uninfected and sucrose-fed (sucrose control, *n* = 10) and uninfected and water-fed (uninfected control, *n* = 10).	Hyperglycaemia	Severity of *tuberculosis* infection in non-diabetic guinea pigs	The exacerbation of insulin resistance and hyperglycaemia by Mtb infection alone may explain why TB is more severe in diabetics with poorly controlled hyperglycaemia compared to non-diabetics and patients with properly controlled blood glucose levels.
J. Peter Cegielski	2012	A prospective study of 13,419 adults from 25 to 72 years of age	different levels of nutritional status	Incident cases of TB	Population's nutritional profile is an important determinant of TB incidence, after controlling for socio-economic factors, excess alcohol consumption, smoking, and DM
Yung-Feng Yen	2016	Population-Based Follow-Up Study of 1608 patients	(<18.5), normal (18.5–24.9), and overweight (≥25).	TB treatment outcome	Insufficient body weight was associated with higher risks of TB-specific and non-TB-specific mortality during TB treatment, particularly in male patients.

The studies included in this review revealed the following outcomes:

### Synergistic effects between TB, immunosenescence and DM

Immunosenescence and DM act synergistically to limit macrophage activation, which in turn decreases IL 12 and consequently IFN gamma, which is believed to play a central role in CMI against intracellular infection primarily by acting on Natural Killer (NK) and T cells. This is achieved through the following mechanisms:

#### Mononuclear phagocytes

Activated mononuclear phagocytes stimulate granuloma formation in response to infection. As humans age, the macrophage capacity for phagocytosis diminishes, which is why the oxidative burst is compromised in elderly persons [30]. In a study involving TB patients, alveolar macrophages were less activated and had lower hydrogen peroxide production in those with DM comorbidity [31].

Additionally, in aged individuals the up-regulation of the major histocompatibility complex (MHC) class I and II expression as well as the antigen presentation capacity are reduced in dendritic cells, [32] which as a corollary diminishes interleukin 2 production and reduces T-cell proliferation. DM has been shown to hamper receptor-bound material, [33] which further limits the role of antigen-presenting cells in lymphocyte activation by preventing the phagocytes from binding and internalizing the antigen, for processing and presentation via their Fc receptors.

#### Natural Killer (NK) cells

It is well known that in elderly humans NK cells show diminished cytotoxic capacity on a 'per cell' basis [34]. Other aspects of NK cell function, such as the secretion of IFN-γ in response to IL-2 and IL 12 are also compromised in the aging population [35]. The decrease in IFN-γ produced by NK cells is further emphasized in individuals with concomitant DM.

#### T lymphocytes

As humans age, the thymus naturally atrophies and the ability of stem cells to undergo clonal proliferation declines. This further emphasizes the reduced secretion of IL IFN gamma by the macrophages and NK in ageing individuals with DM. The inability to produce adequate numbers of mature T lymphocytes compromises the ability of elderly individuals to respond effectively to infections.

As T cells age, they also lose their capacity to produce and respond to IL-2 and IL 12 as major inducers of Th1 type responses, resulting in increased susceptibility to bacterial and viral infections and neoplasias among elderly persons, compared to young adults [36]. An additional synergistic factor is the impaired host resistance in individuals with DM. Namely, lymphocyte proliferation in response to phytohaemagglutinin has been found to be weak in patients with poorly controlled DM [37].

Resistance to mycobacterial infections is mediated largely by T helper type 1 (Th1) cells and their cytokines, whilst Th2 cells and their cytokines correlate with disease susceptibility and pathology in TB [38]. Whilst Th1 cytokines induces Th1 activity and block Th2 activity, [39, 40] Th2 cytokines promote Th2 activity while inhibiting Th1 activity [41]. The decrease in Th1:Th2 ratio may be of great importance in age-related immune changes, since Th1 mainly induces maturation and activation of the cytotoxic T lymphocytes which decrease with ageing, [42] while Th2 induces increased B lymphocyte immunoglobulin production which increases with ageing [43]. A recent study showed that diabetic TB patients had lower Th1:Th2 cytokine ratios and a higher Th2 bias. Also, the concentration of IL-4 alone was significantly higher in diabetic TB patients compared to non-diabetic TB patients and healthy subjects. As higher concentration of IL-4 is also found in aged individuals due to dysregulation between Th1 and Th2, [44] this synergistic elevation in IL-4 secretion may contribute to increased pathogenesis in diabetic TB patients because IL-4 impairs anti-microbial activity of infected cells and increases availability of iron to intracellular *M. tuberculosis*.

Elderly DM patients also show an altered T cytokine production pattern. In aged individuals, lymphocytes produce less IFN-γ, the main T-helper-1 (Th1) cytokine and the main mediator of protective immunity against TB. DM in aged individuals might further adversely affect T-cell production of interferon γ, especially in high-glucose conditions. Consequently, T cell growth, function and proliferation is affected, further compromising effective mononuclear phagocytes and NK cell mediated response to tuberculosis.

Studies show that when the glycated hemoglobin (HbA1c) is <8.0 %, the proliferative function of CD4 T lymphocytes and their response to antigens remains unimpaired [14]. This was also illustrated in a study of 4690 elderly diabetic patients in Hong Kong: those with haemoglobin A1c > 7 % had a three times higher risk of active tuberculosis compared with those with haemoglobin A1c < 7 % (HR 3 · 11; 95 % CI 1 · 63–5 · 92) [45].

In Taiwan, a prospective study of 17 715 Taiwanese persons selected from the general population suggested that the participants’ risk of tuberculosis increased as the number of complications of DM increased (*P* = 0.0016), with >3-fold risk among those with ≥2 diabetes-related complications (odds ratio, 3.45; 95 % CI, 1.59–7.50) [46].

Aside from clinically manifested diabetes, a study on infected guinea pigs showed that non-diabetic hyperglycaemia has the potential to significantly worsen active TB, [47] suggesting that inflammation-associated insulin resistance during *TB* infection may be an additional factor contributing to hyperglycaemia.

#### Synergistic interactions between TB, ageing and nutritional status

The lower disease-fighting capacity of the elderly, especially diabetics, which is partly attributable to the deregulation of the immune system and the greater secretion of macrophage pro-inflammatory cytokines in response to antigenic challenge, also leads to greater or longer-lasting body metabolic changes in this population group [48].

Whilst no change was found in T cell function in very healthy elderly individuals without nutritional deficit,[48] micronutrient deficiency has been shown to further lower immunity by affecting all parameters of CMI, well beyond the effect attributable to the ageing process alone, resulting in increased susceptibility to infectious diseases [49]. The decrease in immune functions strongly correlates with the level of nutritional deficiency, [50] with severely immuno-deficient aged individuals suffering from severe PEM, a condition marked by insufficient protein and calorie intake. Whilst PEM significantly affects innate immunity in the elderly, [51] it chiefly impairs CMI. The resultant atrophy of the thymus [52] reduces the number of circulating T cells, thereby decreasing the effectiveness of the memory response to antigens. PEM is also associated with significantly reduced vaccine antibody responses in the elderly population [53].

The link between body weight and TB has been increasingly reported in scientific literature. A systematic review from 2009 found a strong and consistent log-linear relationship between TB incidence and BMI across a variety of settings, with different levels of TB burden [54]. In populations where protein insufficiency is common, it may contribute substantially to TB incidence [55]. Namely, a recent large prospective study showed that the population-estimated hazard of developing TB for persons with low BMI (<18.5 kg/m^2^) was HR 12.4 (95 % CI: 5.7, 26.9) greater compared to persons with normal BMI, after controlling for socio-economic factors, excess alcohol consumption, smoking and diabetes mellitus [28].

Also, a cohort study in Taiwan involving patients with a mean age of 64.6 years found that insufficient body weight was associated with higher risks of TB-specific and non-TB-specific mortality during TB treatment, particularly in male patients, after adjusting for age, sex, clinical findings, and comorbidities [56].

#### Synergistic association between DM and PEM

Insulin is a strongly anabolic hormone for protein, [57] fat, [58] and glycogen [59] accrual, and deficiency or resistance to insulin may also promote PEM. In addition, comorbidities that diabetic patients are more prone to, such as catabolic events including myocardial infarctions, strokes, infection, ischemic atrophy, [60, 61] cutaneous ulcers and gangrene [62] in the extremities increase the likelihood of PEW/ PEM. Studies have demonstrated a higher frequency of protein-wasting in diabetic patients compared to nondiabetic end-stage renal disease patients on maintenance dialysis therapy [63–65].

It is noteworthy that ageing is also associated with decreased capacities to cope with metabolic changes resulting from underfeeding and/or nutritional responses to disease [66]. A personal communication by Nyadzayo-Rossi (who carried out a not yet published cohort study in moderately undernourished adults in Zimbabwe) found that moderately undernourished adult TB patients appear to be at a higher risk of not responding to nutritional treatment compared to those not affected by TB. This finding highlights the need for preventing undernutrition in aged individuals in order to improve TB control, while considering the impact on glycaemic control.

PEM in turn contributes to increased frailty due to depletion of protein body reserves and consequent susceptibility to recurrent TB episodes or other infections in these individuals, already suffering from a lower protection against free radicals. Consequently, PEM is compounded in aged individuals, who suffer from reduced immune responses [49]. Moreover, there is also a possibility of increased TB drug malabsorption in elderly patients [67]. This may be accentuated by PEM, which in turn impairs drug absorption, [68] thereby adding a new dimension to the growing problem of MDR TB. In 2011, a meta-analysis estimated that DM patients have a risk ratio (RR) for the combined outcome of failure and death of 1.69 (95 % CI, 1.36 to 2.12) and an increased risk of relapse (RR, 3.89; 95 % CI, 2.43 to 6.23) after TB treatment [69]. Several studies have reported a high prevalence of DM among patients with MDR-TB, [70–72] including an association between DM and MDR-TB after adjusting for confounding factors [73]. However, 4 studies in disparate settings showed no significant increased risk [74–76].

According to WHO estimates, less than 50 % of all cases worldwide are currently diagnosed and treated [77]. Furthermore, due to compromised immunity, such as HIV infection, atypical clinical and radiological features in elderly individuals with DM and PEM as well as false negative tuberculin reactions may be very frequent [78]. A recent study underscored how untimely and difficult diagnosis, along with a higher risk of poor outcomes even after treatment completion emphasizes the need to devise specific strategies for this vulnerable group [79].

#### TB drug management in diabetic patients

Diabetes negatively affects TB drug treatment, especially in patients with poor glycaemic control, which may be attributed to altered drug pharmacokinetics in DM. DM affects protein, lipid and carbohydrate metabolism as well as various aspects of pharmacokinetics. These include changes in absorption for several drugs administered via the subcutaneous and intramuscular route, as well as the oral route due to disordered gastric emptying (generally abnormally slow in 30–50 % DM patients, also slower during hyperglycaemia and accelerated during hypoglycaemia) [80]. Furthermore, the impact of DM on enzymes/transporters involved in drug biotransformation, as well as the reduction of plasma protein binding and displacement of drugs from their protein binding sites (due to higher circulating amount of free fatty acids in DM) [76] also affects drug pharmacokinetics. A recent study on TB showed that disease progression in guinea pigs with impaired glucose tolerance was similar to that of non-diabetic controls in the early stages of infection but got more severe by day 90 [81]. Drug toxicity risk may be higher due to excessive drug accumulation in the body, as a result of diabetic nephropathy.

A study involving 17 Indonesian patients with TB-DM comorbidity found 53 % lower plasma concentrations of rifampicin in these patients, compared to patients with TB only. These pharmacokinetic changes have been associated with clinical failure and acquired drug resistance [82]. These observations are congruent with those of a study from Mexico published in 2013 which reported that the proportion of TB patients on first line TB therapy who converted sputum cultures to negative ≥60 days of treatment was significantly greater in patients with DM (45.9 %) compared to those without DM (37.2 %) [83].

#### MDR TB drug management in diabetic patients

The influence of DM on MDR-TB patient outcomes is understudied [84]. Findings from a study on MDR-TB in 2012 involving diabetic patients from five countries, reported a lower non-significant univariate rate ratio (HR 0.76, 95 % CI 0.54–1.06) of sputum culture conversion among patients with DM [85]. These findings are congruent with those of a recent cohort study of adult pulmonary MDR-TB patients from Georgia, according to which, after adjusting for important confounding factors, the rate of sputum culture conversion and the risk of poor treatment outcome was similar in MDR-TB patients with and without DM, adjusted HR estimate (0.95, 95 % CI 0.71–1.28).

A bi-directional relationship has also been observed, as ethionamide or protionamide have been shown to make DM management in patients undergoing treatment with the above drugs more difficult [66].

### Research gaps

#### Association between DM and PEM in the elderly population

Randomized clinical trials exploring the optimal methods of monitoring for PEM as well as prevention and treatment of these disorders in diabetic elderly patients are clearly needed.

#### Association between DM and MDR TB

At a biological level, there is a need of a better understanding of diabetes-related immunopathogenic mechanisms reinforcing immunosenescence, and mechanisms by which DM may lead to MDR TB.

### The impact of DM on TB and MDR TB treatment outcomes

Despite extensive research efforts to elucidate the effects of DM on pharmacokinetics and pharmacodynamics and the significant progress made in this area, the available information is nonetheless insufficiently clear. Additional clinical studies are needed to fully unravel the clinical significance of these effects. DM - mediated changes in the pharmacokinetics of a particular drug must not be generalized to include other drugs, as changes are drug-specific.

Biological mechanisms potentially contributing to TB and MDR TB treatment failure as well as those interfering with glucose control during rifampicin and ethionamide therapy must be elucidated in conjunction with further epidemiological studies exploring the impact of DM on both first line and second line treatments.

In contrast to experimental animal models, DM-related effects on pharmacological properties of drugs in human subjects have not been sufficiently elaborated. Further larger prospective research aimed at understanding the diabetes-mediated changes as well as the sources of the variability is required to help optimize drug management and improve clinical outcomes in DM patients [86].

Furthermore, in order to avoid future discrepancies between individual clinical studies, as well as between ex vivo and clinical studies on this issue, due attention should be paid to adequate selection of test subjects, the type, severity and duration of the disease, histopathological characteristics, as well as other important factors such as medication use, protein intake, age, sex and obesity.

#### Association between low BMI and TB

Although a large prospective study found that persons with low BMI (<18.5 kg/m^2^) are at higher risk of developing active TB compared to those with normal BMI, after controlling for socio-economic factors, excess alcohol consumption, smoking, and diabetes mellitus, it may also be useful to adjust for micronutrient deficiencies. Also, this area of research would benefit from a case control study, wherein active TB elderly patients are compared to non active TB patients in terms of BMI, using the WHO BMI categorization [87]: <16 = severe malnutrition, 16–16.99 = moderate malnutrition, 17–18.49 = mild malnutrition; 18.5-24.99 = normal weight, 25–29.99 = overweight; ≥30 = obese.

Furthermore, it may also be necessary to explore more thoroughly whether TB patients have more difficulties in recovering from malnutrition compared to non-TB patients, a recommendation which is also stipulated in the WHO nutritional guidelines for TB [88].

#### Clinical diagnostic algorithm for elderly patients with DM

Reducing the duration of infectiousness will require establishment of an appropriate clinical diagnostic algorithm and adaptation of clinical guidelines to address elderly patients with DM. Clinical research is warranted to establish a clinical diagnostic algorithm as well as clinical management guidelines targeting this population.

#### Prevention of active TB

Twenty randomized trials involving treatment of latent TB infection (LTBI) to prevent active TB in more than a dozen countries demonstrated that isoniazid (INH) administered for at least 6 months in persons with LTBI reduced subsequent TB incidence by 25 to 92 % [89]; the differences were mainly attributed to variation in treatment completion. In the elderly population with DM and PEM, the effectiveness of an INH regimen still needs to be determined, along with exploring its high potential for hepatotoxicity and the pharmaco-epidemiology of INH interaction with other drugs.

#### Bi-directionality of TB and DM

Studies have shown that TB can even cause DM in those not previously known to be diabetic, with TB patients showing higher rates of glucose intolerance than community controls [33, 90, 91]. This may not only lead to a global explosion of TB cases, but also DM cases. Furthermore, determining the bi-directionality of TB and DM and the potential of DM to fuel not only a TB, but potentially a MDR TB epidemic will need to be investigated.

## Discussion

At a global level, the lethal cocktail of ageing population with PEM, latent TB and concomitant diabetes mellitus sets the stage for increased TB transmission that may fuel a TB epidemic and possibly a MDR TB epidemic.

Reducing TB transmission in low, middle and high income settings will require a multipronged approach. In middle and higher income countries, the increasing number of people residing in nursing and chronic disease facilities places residents at greater risk for developing active TB than elderly persons living in the community, with the potential of spilling over within the community. In low-income countries, higher life expectancy combined with an already high prevalence of PEM may fuel an unprecedented TB and possibly an MDR TB epidemic.

This begs for an integrative framework for enhanced TB control and prevention among aged diabetics as well as a stringent screening of glycaemic responses in TB patients at the time of diagnosis, even if diagnostic criteria for diabetes are not met [84, 92] This integrative framework should be tailored to the contexts of industrialized and middle - income countries. To this end, the working definition of “old” must first be established per setting. Most industrialized nations have accepted the chronological age of 65 years as a definition of 'elderly' or older person, [93] whilst in sub-Saharan Africa a MDS/WHO established the age of 50 years as a working definition of “old.” This will be all the more relevant for low- to middle-income countries, including India and China, which have the highest burden of TB and are experiencing the fastest increase in diabetes prevalence [16].

As an adjunct, PEM management should be strengthened. PEM management research must also encompass a better appreciation of the nutritional status of the elderly population, especially in resource-poorer countries. In addition, another salient area of research would be to explore the protective effect of higher BMI in preventing development of active TB or improving disease outcome. If these observations are confirmed, policies regarding isoniazid preventive treatment could be revisited because the potential benefit would be much lower among elderly persons with medium to high BMI. Moreover, WHO guidelines for TB nutritional support must establish a calorie intake threshold for aged diabetic patients.

## Conclusion

The still poorly elucidated tripartite association involving TB, PEM, and DM in the ageing population threatens to place a major burden on the public health system. A number of epidemiological and clinical gaps must be addressed to curb transmission. Enhanced PEM management, especially in resource-poorer settings, the development of a clinical diagnostic algorithm as well as clinical guidelines for this population are urgently needed. To thwart an explosion of TB and potential MDR TB and DM epidemics, the transmission dynamics should be examined.

## Abbreviations

BMI, Body Mass Index; DM, Diabetes Mellitus; IFN-γ, Interferon gamma; LTBI, Latent TB infection; MDR TB, Multi drug resistance TB; PEM, Protein Energy Malnutrition; TB, Tuberculosis
